# Efficacy of Mudan Granule (Combined With Methylcobalamin) on Type 2 Diabetic Peripheral Neuropathy: Study Protocol for a Double-Blind, Randomized, Placebo-Controlled, Parallel-Arm, Multi-Center Trial

**DOI:** 10.3389/fphar.2021.676503

**Published:** 2021-05-20

**Authors:** Yuehong Zhang, De Jin, Yingying Duan, Rui Hao, Keyu Chen, Tongyue Yu, Fengmei Lian, Xiaolin Tong

**Affiliations:** ^1^ Department of Endocrinology, Guang’anmen Hospital, China Academy of Chinese Medical Sciences, Beijing, China; ^2^ School of Traditional Chinese Medicine, Beijing University of Chinese Medicine, Beijing, China

**Keywords:** mudan granule, Chinese patent medicine, type 2 diabetic peripheral neuropathy, efficacy, study protocol

## Abstract

**Background:** Diabetic peripheral neuropathy (DPN) characterized by nerve damage is a common and disabling chronic microvascular complication in patients with type 2 diabetic mellitus (T2DM), affecting at least half of patients diagnosed with T2DM. Unfortunately, the current treatment for DPN is not ideal. Traditional Chinese medicine (TCM), with a unique theoretical system, has made outstanding contributions in the treatment of T2DM and related complications. Mudan granule, a Chinese patent medicine, has been previously validated that could ameliorate the symptoms, promote nerve tissue repair, increase nerve conduction velocity (NCV) in patients with DPN. However, the previous studies are of variable quality, which limits the clinical application of Mudan granule. Therefore, we designed a double-blind, randomized, placebo-controlled, parallel-arm, multi-center trial to estimate the safety and efficacy of Mudan granule in conjunction with methylcobalamin in individuals suffering from type 2 diabetic peripheral neuropathy.

**Methods:** This work is conducted as a 14-center, double-blind, randomized, placebo-controlled, parallel-arm trial. In all, 402 subjects (aged 30–70 years) will be recruited and randomized in a 1:1 ratio to an intervention group (*n* = 201; Mudan granule + methylcobalamin) and a control group (*n* = 201; placebo + methylcobalamin). Treatment is administered in 24 weeks cycles without any treatment interruption between cycles. Michigan Diabetic Neuropathy Score (MDNS) as the primary outcome will be evaluated at baseline, 12 weeks during the intervention period, and after 24 weeks of the intervention. Adverse events and safety assessments will be also documented. The analysis of all data will be carried out based on a predefined statistical analysis plan.

**Discussion:** The outcomes from this study will offer important evidence regarding the safety and efficacy that Mudan granule can be used as an alternative and complementary therapeutic intervention in patients with type 2 diabetic peripheral neuropathy.

**Clinical trial registration:** Registered at https://clinicaltrials.gov/. Trial registration number: NCT04711980. Registered January 2021.

## Introduction

Type 2 diabetes mellitus (T2DM), caused by metabolic disturbances, is often linked to long-term complications and has adverse effects on the eyes, kidneys, heart, and nerves (Bergrem and Leivestad, 2001; Chen et al., 2011; Yau et al., 2012; Albers and Pop-Busui, 2014; Zheng et al., 2018), seriously impacting global health. In accordance with the International Diabetes Federation update of 2019 (Saeedi et al., 2019), around 90% of the 463.0 million individuals aged 20–79 worldwide who were affected by diabetes suffered from T2DM in 2019. The global prevalence of T2DM has significantly contributed to a corresponding epidemic of complications. Among the complications, Diabetic peripheral neuropathy (DPN) is the most frequent and disabling chronic microvascular complication in patients with T2DM, presented clinically with loss of sensation, tingling, pain, hyperalgesia, and weakness (Peltier et al., 2014; Callaghan et al., 2020). In addition, DPN significantly contributes to the increased morbidity with diabetic foot ulcers and amputation (Tesfaye et al., 2016; Megallaa et al., 2019), which not only inflicts devastating suffering on individuals and their families but also emerges as a major cause for loss of physical independence and potential years of life (Chammas et al., 2016).

Without successful interventions, impacts on the individual, families and even the whole society would be disastrous. At present, glycemic control and symptomatic relief serve as a cornerstone in the treatment for patients with type 2 diabetic peripheral neuropathy (Hemmingsen et al., 2013; Alam et al., 2020). Although tricyclic antidepressants, serotonin–noradrenaline reuptake inhibitors, and anticonvulsants that act on calcium channels are recommended as first-line treatment (Khdour, 2020 ), its therapeutic management remains challenging. Adverse reactions include fatigue, somnolence, dizziness, and peripheral edema are inevitable (Boyle et al., 2012; Author Anonymous, 2015). Therefore, additional therapeutic strategies are required to combat this disease.

Integrative medicine that combines conventional Western medicine withtraditional Chinese medicine (TCM) has gradually become the main approach to treat many diseases. As a vital branch of TCM, Chinese patent medicines have been extensively applied in the clinical field as derivatives of Chinese herbal medicine (Ng et al., 2012; Chen et al., 2014). Chinese patent medicines frequently used alone or in conjunction with western medicine could successfully lower blood glucose levels and relieve symptoms in patients with diabetes and diabetic complications (Lian et al., 2015; Lian et al., 2015).

A clinical study confirmed that Chinese patent medicine (Xuesaitongcapsule) combined with *α*-lipoic acid was effective and safe in the treatment of DPN with qi-deficiency and blood stasis, while also reduced the expression of TNF-α and IL-1β in serum (Zhang and Zhu, 2020). Moreover, a network Meta-analysis evaluating the efficacy and safety of the blood-activating stasis-removing Chinese patent medicines for DPN showed that some Chinese patent medicines combined with methylcobalamin were superior to the treatment of the general and methylcobalamin groups in the sensory and motor conduction velocity of the median nerve (Zuo et al., 2020). Mudan granule, a Chinese patent medicine, consisting of Huangqi (*Astragalus*), Yanhusuo (*Corydalis*), Sanqi (*Panax notoginseng*), Chishao (*Radix paeoniae rubra*), Danshen (*Salvia miltiorrhiza*), Chuanxiong (*Ligusticum chuanxiong hort*), Honghua (*Carthamus tinctorius*), Sumu (Logwood), and Jixueteng (*spatholobi Caulis*) has been investigated to be beneficial clinically and experimentally in the treatment of DPN (Wu, 2021; Li, 2020). One of the clinical studies with 60 subjects showed that Mudan granule significantly improved 2 h postprandial blood glucose, glycated hemoglobin, and Michigan Neuropathy Screening Instrument (MNSI) score (Xun and Liu, 2020). Notably, overall the studies of Mudan granule available have involved small sample sizes, short duration, poor methodological quality, and without clinically registered information. For these reasons, high-quality clinical evidence of the safety and efficacy of Mudan granule in individuals with type 2 diabetic peripheral neuropathy is required urgently. Our research team will provide such a high-quality randomized controlled trial (RCT) study. This study aims at presenting the methodologies and full details of the protocol.

## Methods/Design

### Study Objectives

The trial is conducted for assessment of safety and efficacy of Mudan granule with a combination of methylcobalamin in improving Michigan Diabetic Neuropathy Score (MDNS) and relieving clinical symptoms in patients with type 2 diabetic peripheral neuropathy. We presume the hypothesis that Mudan granule + Methylcobalamin is more potent and efficient than Placebo + Methylcobalamin in improving MDNS and alleviating the clinical symptoms of people with type 2 diabetic peripheral neuropathy. 402 clinically stable patients will be randomized into this trial within the 24 weeks period. Recruitment performs in out-patient clinical institutions.

### Study Design

**FIGURE 1:**
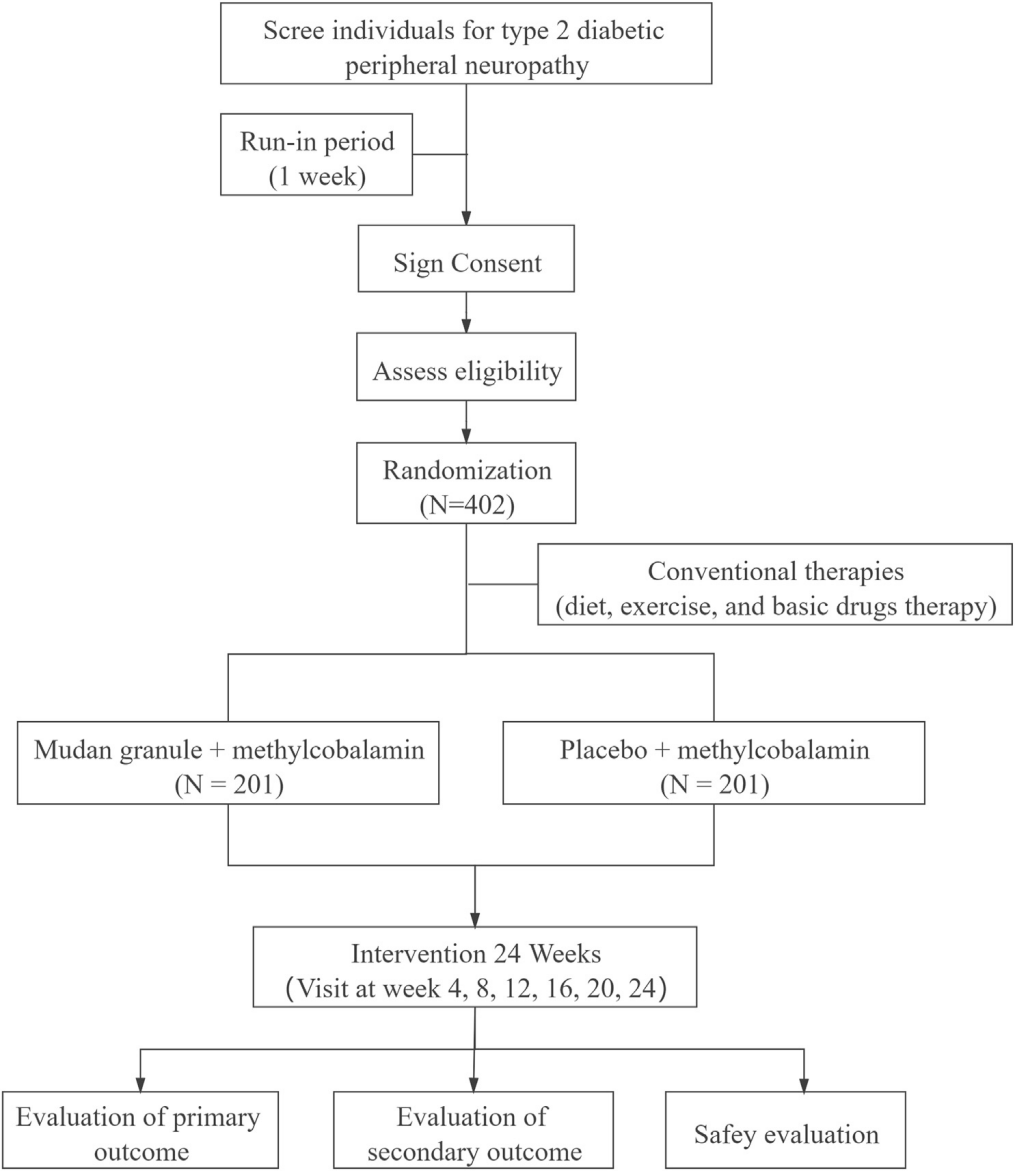
The flow diagram of this study.

To test our hypothesis, a double-blind, randomized, placebo-controlled, parallel-arm, multi-center trial is designed. Fourteen research cooperation centers in China will participate in the trial, including Peking Union Medical College Hospital, Beijing Hospital, Affiliated Hospital of Liaoning University of Traditional Chinese Medicine, Affiliated Hospital of Qingdao University, Chengdu University of Traditional Chinese Medicine, The Fourth People’s Hospital of Chongqing, Zhengzhou Yihe Hospital Affiliated to Henan University, Zhu Xianyi Memorial Hospital of Tianjin Medical University, The Third Hospital of Xi’an, Huashan Hospital Affiliated to Fudan University, Gansu Provincial People’s Hospital, Shenzhen Hospital of Guangzhou University of Traditional Chinese Medicine, and Affiliated Hospital of Changchun University of Chinese Medicine. This clinical trial was registered with clinicaltrials.gov; Registration Number for Clinical Trial: NCT04711980; Any changes related to the protocol will be presented there. Figure 1 displays the flow diagram of this study.

### Inclusion Criteria

1. Meet the diagnostic criteria of type 2 diabetic peripheral neuropathy (Author Anonymous, 2018):
(a). Have a history of T2DM.

–The definition (American Diabetes Association, 2021) of T2DM is that fasting plasma glucose ≥126 mg/dl (7.0 mmol/L)

- Fasting refers to no caloric intake for at least 8 h*

OR.

- Two-hour plasma glucose ≥200 mg/dl (11.1 mmol/L) during an oral glucose tolerance test. The test should be conducted as described in accordance with the World Health Organization using a glucose load containing the equivalent of 75 g of anhydrous glucose dissolved in water.*

OR.

- HbA1c ≥ 6.5% (48 mmol/mol).

The test should be conducted in a laboratory using a method that is National Glycohemoglobin Standardization Program-certified and standardized to the Diabetes Control and Complications Trial assay.*

OR.

- In patients with typical symptoms of hyperglycemia or hyperglycemic crisis, a random plasma glucose ≥200 mg/dl (11.1 mmol/L).*

* without the presence of unequivocal hyperglycemia, the examination needs to be repeated twice.

(b). Neuropathy occurs at or after the diagnosis of T2DM.
(c). The presence of symptoms and signs should be aligned with the manifestations of DPN.
(d). In patients with clinical symptoms (pain, numbness, paresthesia, etc.) of DPN, necessitating the need for abnormalities in one or more of the five examinations (stress perception, vibration perception, temperature perception, pain and ankle reflex); In patients without clinical symptoms of DPN, necessitating the need for abnormalities in two or more of the five examinations (stress perception, vibration perception, temperature perception, pain and ankle reflex).
(e). All those with neuropathy resulting from other causes must be excluded.
2. Meet the diagnostic criteria of TCM syndrome of Qi deficiency and collaterals obstruction syndrome.

Diagnostic Criteria of Qi deficiency and collaterals obstruction syndrome (Zheng, 2002):

(a). Primary symptoms and signs: ① numbness; ② pain; ③ paresthesia
(b). Secondary symptoms and signs: ① scaly dry skin; ② dim complexion; ③ fatigue; ④ spiritlessness and talking laziness; ⑤ spontaneous sweating
(c). Tongue condition: light-dark/petechiae tongue, with thin and white coating.
(d). Pulse condition: thin and astringent pulse.

Patients with two or three primary symptoms, two or more secondary symptoms, and tongue pulse signs could be diagnosed as Qi deficiency and collaterals obstruction syndrome.

3. The patients are aged 30–70 years.
4. Conduction velocity achieved using electromyography assessments in 2 or more nerves have decreased.
5. The patient provides signed informed consent.

### Exclusion Criteria

1. Patients have recently taken antioxidants such as vitamin E or vitamin C; acute infection; renal or hepatic insufficiency; acute complications of T2DM; serious cardio-cerebrovascular diseases; neuropathy due to long-term alcohol intake and other factors.
2. Besides methylcobalamin, drugs such as alpha lipoic acid, epalrestat, VitB12, Chinese patent medicine and decoction have been used to treat DPN within 4 weeks before enrollment.
3. Poor control of blood pressure (systolic blood pressure greater than160 mmHg or diastolic blood pressure greater than 100 mmHg).
4. Suffering from diabetic ketosis, ketoacidosis and serious infection within one month.
5. Patients with comorbidities such as cardiovascular, renal, hepatic, and hematopoietic system and other severe primary diseases; serum transaminase over the normal value more than 2 times; serum creatinine greater than the upper limit of normal; psychiatric disorders.
6. Pregnancy, ready to be pregnant or lactating female patients.
7. With a history of multiple drug allergies or being allergic to the ingredients of Mudan granule.
8. Participate in other clinical studies within one month.
9. Alcohol abuse and/or taking psychoactive substances, drug abusers and dependents over the last five years.
10. With a history of active ophthalmopathy, ocular surgery, glaucoma, acute or chronic corneal diseases, extended-wearing contact lens and so on.

### Withdrawal Criteria

#### The Withdrawal Decided by the Researcher

The researcher can decide to withdraw a subject from the study under the following circumstances:

1. During the study, the subjects may develop severe acute or chronic complications or special physiological changes (e.g., human chorionic gonadotropin test is positive) that not appropriate for this study.
2. During the study, the subjects may have poor adherence to medications, such as failure to take less than 80% or more than 120% of the prescribed dosage.
3. Other treatment drugs were added without following the researcher’s guidance during the entire study period.

### Subjects Withdrawal at Their Own Will

Based on the informed consent form, the subjects are free to withdraw from the trial at any point. Subjects who do not formally withdraw from the trial but no longer receive drugs and undergo testing or who are lost to follow-up are also regarded as withdrawn. The reasons for the subjects withdrawing should be ascertained and recorded wherever possible.

### Intervention Measures

All included subjects will accept conventional therapies containing 0.5 mg oral methylcobalamin tablets three times per day, lifestyle interventions, and other oral drugs to achieve stabilization of blood sugar, blood pressure, and lipids depending on American Diabetes Association guidelines. Subjects will be randomly allocated to either the intervention group (Mudan granule 7 g/bag, three times per day) or the control group (placebo 7 g/bag, three times per day) through a central randomization system. Treatment will continue for 24 weeks.

### Study Procedure

**FIGURE 2:**
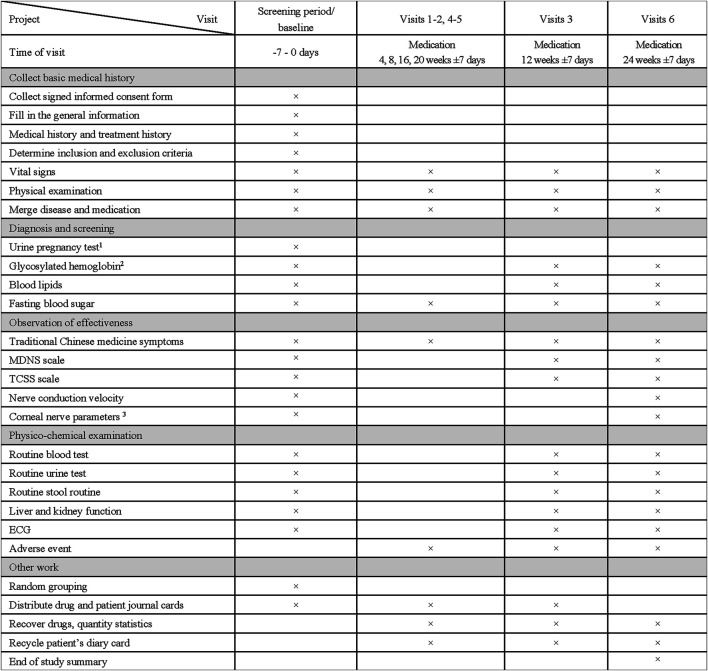
Clinical Study Visit Form. 1 Only for childbearing-age women and secondary amenorrhea women for more than 1 week; Test should be initiated within 24 h prior to the first medication. 2 Blood sample of the glycosylated hemoglobin will be collected and placed in –80°C refrigerator. 3 Only 120 subjects in four centers (Peking Union Medical College Hospital, Beijing Hospital, Affiliated Hospital of Qingdao University, Huashan Hospital Affiliated to Fudan University) will receive this test.

The specific measurements and time points for data collection of this study are outlined in Figure 2. Eligible subjects will undergo a 1-week preintervention screening period and a 24 weeks treatment period. After the research commences, all subjects enrolled will be followed up every 4 weeks for 24 weeks. All subjects will be informed about the oral and written information about this trial and asked to sign informed consent forms.

## Outcome Measure

### Demographic Information

Demographic information such as gender, age, course of disease, previous medical history will be collected.

## Efficacy Outcomes

### Primary Outcomes

Changes in total score on the MDNS Scale: The changes of MDNS will be compared in the two groups prior to and following Mudan granule administration. The total score is 46, ranging from 0 (least severe) to 46 (most severe). Measurement will be performed at baseline and week 12, 24.

### Secondary Outcomes

1. Changes in NCV: The changes in NCV of each nerve will be compared in the two groups prior to and following Mudan granule administration. Measurement will be performed at baseline and week 24.
2. Changes in corneal nerve fiber density of each mm^2^: The changes in corneal nerve fiber density of each mm^2^ will be compared in the two groups prior to and following Mudan granule administration. Measurement will be performed at baseline and week 24.
3. Changes in corneal nerve branch density of each mm^2^: The changes in corneal nerve branch density of each mm^2^ will be compared in the two groups prior to and following Mudan granule administration. Measurement will be performed at baseline and week 24.
4. Changes in corneal nerve fiber length of each mm^2^: The changes in corneal nerve fiber length of each mm^2^ will be compared in the two groups prior to and following Mudan granule administration. Measurement will be performed at baseline and week 24.
5. Changes in total score on the Toronto Clinical Scoring System(TCSS) Scale: The changes of toronto clinical score will be compared in the two groups prior to and following Mudan granule administration. The total score is 19, ranging from 0 (least severe) to 19 (most severe). Measurement will be performed at baseline and week 12, 24.
6. Changes in total score on the TCM Syndromes Efficacy Score Scale: The changes in TCM syndromes efficacy score will be compared in the two groups prior to and following Mudan granule administration. TCM syndromes efficacy score is 45, ranging from 0 (least severe) to 45 (most severe). Measurement will be performed at baseline and week 4,8,12,16,20,24.
7. Changes in total score on the Clinical Symptoms Score Scale: The changes of each clinical symptom score will be compared in the two groups prior to and following Mudan granule administration. Clinical symptom score ranging from 0 (least severe) to 6 (most severe). Clinical symptoms mainly included pain, numbness, and paresthesia. Measurement will be performed at baseline and week 12, 24.

### Monitoring Outcomes

1. Fasting blood glucose, and blood pressure. Measurement will be performed at baseline and week 4,8,12,16,20,24.
2. Glycated hemoglobin, and blood lipid. Measurement will be performed at baseline and week 12, 24.

### Safety outcomes and adverse events.

1. Adverse events: adverse events will be monitored for 6 months from the time the subjects enrolled in the study to the last follow-up. All information about adverse events will be carefully recorded at any time, such as their intensity, duration and relationship with the Mudan granule. The intensity of adverse events will be categorized as light, moderate and heavy. Serious adverse events must be reported immediately to the research center, the main research medical ethics committee, and the primary sponsor. At the same time, it will be reported to the National Medicinal Product Administration (NMPA) within 24 h. In addition, a Serious Adverse Event Form must be completed as soon as possible.
2. General vital signs: body temperature, heart rate, respiration and so on. Measurement will be performed at baseline and week 4,8,12,16,20,24.
3. Blood routine, urine routine, urine pregnancy test, stool routine, 12-lead Electrocardiography, hepatic and renal function tests. Measurement will be performed at baseline and week 12, 24.

### Sample Size Calculation

Depending on the previous study (Li and Yu, 2016) of Mudan granule combined with methylcobalamin in the treatment of DPN with 12 weeks intervention, the results of the MDNS indicated that the study group (Mudan granule + Methylcobalamin) scale score was 5.48 ± 1.19, the control group (Placebo + Methylcobalamin) scale score was 8.21 ± 1.24, and the mean difference between the two groups was 2.73; Assuming that Mudan granule combined with methylcobalamin in the treatment of DPN can reduce the MDNS by 0.58 after 12 weeks of intervention, it is considered more effective than the control group. Taking *α* = 0.05, *β* = 0.10, the intervention group and the control group with 24 weeks intervention were allocated at a ratio of 1:1. The sample size calculated by the Power Analysis and Sample Size (PASS) 11 software is not less than 167 cases in each group. Considering that an expected withdrawal rate does not exceed 20%, the target sample size is set at 402, with 201 cases in each group.

### Randomization

Randomization of this study will be performed as block randomization. Statistical Analysis System Software (SAS) software version 9.4 will be used to generate the blind code of randomization groups with an allocation ratio of 1:1 between the intervention group and the control group. After subjects completing the screen and providing written informed consent forms, the random applicants will apply for a drug number for the subject according to the group to which the subject is randomized via a central randomized system. The drug number of each subject remains the same throughout. If, for some reason, clinical emergencies occur, the personal random code number and random grouping will be made public.

### Blinding

This trial is conducted as a double-blind study. In this double-blind clinical study, researchers, subjects, monitors, statisticians, and data administrators are unaware of the treatment group assignments, which can reduce the bias to the greatest extent. In the event of a clinical emergency, the principal investigator, sponsor, and other people concerned will decide to remove the blindness.

Besides, Mudan granule and placebo granule are indistinguishable in appearance and taste. During the process of placebo preparation, we consider various factors including the properties, production process, prescription and excipients of the original therapeutic drug. Dextrin, pregelatinized starch and microcrystalline cellulose are admixed in varying proportions. As the original therapeutic drug taste bitter, so sucrose octaacetate is added to rectify the taste. The final color results from the addition of food-grade natural caramel pigment. Lastly, Mudan granule and placebo granule are concealed in uniform packages with the same labels.

## Data Collection and Monitoring

### Data Collection

In this study, the Electronic Data Capture (EDC) system will be used for data collection and management. The data collected in the electronic case report form (eCRF) should be consistent with the original medical records, laboratory examination reports and other original documents.

### Design of Electronic Case Report Form

The eCRF should ensure that all data needed for analysis are collected accurately and effectively according to the research protocol.

### Medical Codes

Adverse events will be coded in terms from the Medical Dictionary for Regulatory Activities (MedDRA) (version 22.0) terminology prepared by the International Council for Harmonization (ICH).

### Data Entry and Modification

The data administrators develop the entry instructions/guidelines of the eCRF. All researchers/clinical research coordinators involved in this study will be strictly trained to ensure that the original data is entered into the EDC system accurately, timely, completely and normatively. After data entry, it is not allowed to modify data at will. In cases where there is a need to modify the data, modification reason should be filled in according to the systemic cues. All operations in the system are traceable.

### Blind Review and Database Lock

After the last eCRF entering into the EDC system and all problematic data resolved, the principal researchers, sponsors, statisticians and data administrators will conduct a blind review. Blind review needs to determine which analysis set each case belonging to and how to deal with missing values and outliers.

### Unblinding

After all the data has been verified and locked, the principal researchers, statisticians, and sponsors are responsible for the statistical analysis plan. When removing the blindness for the first time, it will indicate the group to which the patient belongs (only either group A or group B will be assigned to each patient). Any modification to the database is possible only by joint written agreement between the head of clinical researchers, statisticians, and data administrators. The first level blind codes will be handed over to the statisticians for statistical analysis according to the statistical analysis plan. Finally, the statistical analysis report will be developed by the statisticians, and the second unblinding (that is, the patient belongs to the control or intervention group) will be performed at the summary meeting.

### Statistical Analysis

#### Definition and Selection of Statistical Analysis Data Set

The subjects in the trial will be categorized as follows:

1. Full Analysis Set (FAS)

FAS is defined as the data set composed of all subjects who are block randomized and have at least one efficacy evaluation result after baseline, which is the main analysis set for efficacy evaluation. Demographics and other baseline characteristics are also set as the FAS. The missing value of the primary efficacy indicators will be filled with the last observation carried forward. In comparison, the missing values of secondary efficacy and safety indicators will not be filled.

2. Per Protocol Set (PPS)

PPS comprises all the FAS subjects who meet the inclusion criteria but not the exclusion criteria and complete the treatment protocol without seriously violating the study protocol, which is the secondary analysis set for efficacy evaluation.

3. Security data set (Safety Set, SS)

SS includes all subjects who are block randomized, receive at least one dose of Mudan granule, and have at least one postbaseline safety evaluation.

### Statistical Analysis Method

SAS 9.4 will be employed for statistical calculations. All data statistics will be analyzed by using a bilateral test, and *p* ≤ 0.05 suggested statistical significance. Count data will be expressed by the constituent ratio. The description of the quantitative data will use the mean, standard deviation, median, and minimum/maximum values. All indicators will be analyzed using appropriate statistical methods. Comparisons between groups of quantitative data will be performed using the Wilcoxon rank-sum test and group *t*-test. The main efficacy indicators will be described with the diachronic statistical description. Analyses of covariance with baseline as a covariate will be used to adjust for comparisons between groups. If necessary, the center effect may be considered and the least square means (LS-means) with their 95% confidence intervals (95% CI) for each group will be calculated. Chi-squared or Fisher’s exact test will be used for categorical data, while grade data will be analyzed by the CMHχ^2^ test or Wilcoxon rank-sum test.

### Safety Assessment

Adverse events coded using MedDRA prepared by the ICH will be tabulated and described. Comparisons between groups of the incidence of adverse events will be employed by the χ^2^ test or the Fisher exact test. Meanwhile, statistical description of the normal/abnormal changes of laboratory test results before and after administration of Mudan granule in each group and their relationship with Mudan granule will be described in detail.

### Current Status

This trial is not yet recruiting. Potential protocol modifications will be documented at https://clinicaltrials.gov/. Trial registration number: NCT04711980.

## Discussion

Parallel to the increasing global incidence of diabetes, the prevalence of type 2 diabetic peripheral neuropathy is steadily rising, which makes it one of the major health threats and socio-economic burdens in the 21st century. Mudan granule, a Chinese patent medicine, independently developed in China is used to treat DPN. Potential mechanisms of Mudan granule therapy may include improving insulin resistance, reducing apoptosis of islets, neuroprotection, and antioxidant effects (Li and Yu, 2016a; Li and Yu, 2016b; Liu et al., 2014; Li, 2014). Clinical researches have demonstrated that Mudan granule based on the conventional treatments offered by modern medicine may effectively enhance the therapeutic effect of treatment (Wu, 2021; Liu et al., 2020). Unfortunately, the therapeutic application of Mudan granule is limited due to low quality, and quantity, of researches. Thus, an evidence-based high-quality clinical trial to assess the efficacy and safety of Mudan granule in the treatment of DPN has merit.

This research is a 14-center study with a large sample size (402 cases). All outcomes, including TCM syndrome, used in the criteria can be quantified in modern medicine. Based on the TCM syndrome which is with the basic units of symptoms (He and Hiroshi, 2008), the enrollment of patients with Qi deficiency and collaterals obstruction syndrome can contribute to the reduction of potential selection bias. Qi deficiency and collaterals obstruction syndrome mainly manifested as pain, numbness, and paresthesia is a very common syndrome observed in DPN (Zhao et al., 2020; Yang et al., 2020). While Mudan granule approved by the Chinese State Food and Drug Administration, mainly exerts qi-reinforcing, blood circulation-promoting and stasis-removing effects according to TCM theory. The score of TCM syndrome is used to elucidate the objectivity of quantifying TCM symptoms. From another perspective, the integration of TCM syndrome and modern medicine plays out vividly in this research.

However, this study also has certain limitations. Patients age greater than 70 years or younger than 30 years were not included in this research. Indeed, patients over the age of 70 years are more prone to developing more severe DPN. Excluding this population group may be attributed to several reasons as follows: In the course of normal aging, there is a decline in peripheral nerve function. For example, NCV is significantly reduced in older compared with young (Liu, 1983). Additionally, older people have various comorbidities and are hard to measure the exact efficacy and safety of the intervention. Moreover, medication compliance in elderly patients may be lower due to their forgetfulness. Therefore, older patients could not be included in our study, otherwise, the rate of lost-to-follow-up will be too high. The number of patients who are less than the age of 30 suffering from type 2 diabetes is relatively small. This population group rarely develop peripheral neuropathy. Hence we decided to exclude this specific population in the study.

Summarily, this study is the first large-sample multi-center RCT of Mudan granule combined with methylcobalamin against type 2 diabetic peripheral neuropathy. If effective, evidence-based medical research of Mudan granule is available, and simultaneously provides a combination of treatment strategy with integrative Chinese and modern medicine, which can be extensively and successfully used in clinical practice. This protocol is designed to avoid reporting biases and to improve transparency.

### Ethical Issues

This trial has been approved by the Ethics Committee (Guang’anmen Hospital of the China Academy of Chinese Medical Sciences). The approval number is 2019–244-KY. Approval has been obtained from the local institutional review boards at all participating centers.

### Dissemination and Consent for Publication

Results of this RCT will be published in peer-reviewed journals and presented at scientific conferences. All subjects were asked for permission to publish the study results and assured of anonymity and confidentiality.

## References

[B1] American Diabetes Association (2021). 2. Classification and Diagnosis of Diabetes: Standards of Medical Care in Diabetes-2021. Diabetes Care 44 (Suppl 1), S15–s33. 10.2337/dc21-S002 33298413

[B2] Alam U. Sloan G. Tesfaye S. (2020). Treating Pain in Diabetic Neuropathy: Current and Developmental Drugs. Drugs. 80 (4), 363–384. 10.1007/s40265-020-01259-2 32040849

[B3] Albers J. W. Pop-Busui R. (2014). Diabetic Neuropathy: Mechanisms, Emerging Treatments, and Subtypes. Curr Neurol Neurosci Rep. 14 (8), 473. 10.1007/s11910-014-0473-5 24954624 PMC5084622

[B10] Author Anonymous. (2015). Correction: Pharmacologic Interventions for Painful Diabetic Neuropathy. Ann Intern Med. 162 (8), 600. 10.7326/l15-0078-3 25894038

[B11] Author Anonymous. (2018) Guidelines for the Prevention and Control of Type 2 Diabetes in China (2017 Edition). Chinese Journal of Practical Internal Medicine. 38 (04), 292-344. 10.19538/j.nk2018040108

[B4] Bergrem H. Leivestad T. (2001). Diabetic Nephropathy and End-stage Renal Failure: the Norwegian Story. Adv Ren Replace Ther. 8 (1), 4–12. 10.1053/jarr.2001.21711 11172323

[B5] Boyle J. Eriksson M. E. V. Gribble L. Gouni R. Johnsen S. Coppini D. V. (2012). Randomized, Placebo-Controlled Comparison of Amitriptyline, Duloxetine, and Pregabalin in Patients with Chronic Diabetic Peripheral Neuropathic Pain: Impact on Pain, Polysomnographic Sleep, Daytime Functioning, and Quality of Life. Diabetes Care. 35 (12), 2451–2458. 10.2337/dc12-0656 22991449 PMC3507552

[B6] Callaghan B. C. Price R. S. Feldman E. L. (2020). Distal Symmetric Polyneuropathy in 2020. Jama. 324 (1), 90–1. 10.1001/jama.2020.0700 32633792 PMC12206378

[B7] Chammas N. K. Hill R. L. R. Edmonds M. E. (2016). Increased Mortality in Diabetic Foot Ulcer Patients: The Significance of Ulcer Type. Journal of Diabetes Research. 2016, 1–7. 10.1155/2016/2879809 PMC4860228 27213157

[B8] Chen G. McAlister F. A. Walker R. L. Hemmelgarn B. R. Campbell N. R. C. (2011). Cardiovascular Outcomes in Framingham Participants With Diabetes. Hypertension. 57 (5), 891–897. 10.1161/hypertensionaha.110.162446 21403089 PMC3785072

[B9] Chen W. Liu B. Wang L.-q. Ren J. Liu J.-p. (2014). Chinese Patent Medicines for the Treatment of the Common Cold: a Systematic Review of Randomized Clinical Trials. BMC Complement Altern Med. 14, 273. 10.1186/1472-6882-14-273 25074623 PMC4129119

[B12] He RR Hiroshi K (2008). Shanghuo Syndrome in Traditional Chinese Medicine. World Science and Technology. 10 (5), 37–41. 10.1016/S1876-3553(09)60024-7

[B13] Hemmingsen B. Lund S. S. Gluud C. Vaag A. Almdal T. P. Wetterslev J. (2013). Targeting Intensive Glycaemic Control Versus Targeting Conventional Glycaemic Control for Type 2 Diabetes Mellitus. Cochrane Database Syst Rev (11), Cd008143. 10.1002/14651858.CD008143.pub3 24214280

[B14] Khdour M. R. (2020). Treatment of Diabetic Peripheral Neuropathy: a Review. J Pharm Pharmacol. 72 (7), 863–872. 10.1111/jphp.13241 32067247

[B15] Li YY Yu SJ (2016a). The Effect of Mudan Particles on the Oxidative Levels of Spinal Cord of Diabetic Rats. Medical Recapitulate 22 (10), 2070–3. 10.3969/j.issn.1006-2084.2016.10.060

[B16] Li YY Yu SJ (2016b). The Effects of MuDan Granules on Neuron Apoptosis of Dorsal Root Ganglion of STZ-induced Diabetic Rats. Western journal of Chinese medicine 29 (06), 16–8. 10.3969/j.issn.1004-6852.2016.06.006

[B17] Li JT (2014). Effect and Mechanism of Mudan Granule on Islet Cell Apoptosis in Diabetic Rats [master]. Liaoning, China: Liaoning University Of Traditional Chinese Medicine

[B18] Li CH (2020). Effects of Mudan Granules on Nav 1.8 of Sural Nerves in Rats with Painful Diabetic Peripheral Neuropathy. Chinese Journal of Misdiagnostics 15 (04), 184–7.

[B19] Lian F. Tian J. Chen X. Li Z. Piao C. Guo J. (2015). The Efficacy and Safety of Chinese Herbal Medicine Jinlida as Add-On Medication in Type 2 Diabetes Patients Ineffectively Managed by Metformin Monotherapy: A Double-Blind, Randomized, Placebo-Controlled, Multicenter Trial. PLoS One. 10 (6), e0130550. 10.1371/journal.pone.0130550 26098833 PMC4476735

[B20] Lian F. Wu L. Tian J. Jin M. Zhou S. Zhao M. (2015). The Effectiveness and Safety of a Danshen-Containing Chinese Herbal Medicine for Diabetic Retinopathy: a Randomized, Double-Blind, Placebo-Controlled Multicenter Clinical Trial. Journal of Ethnopharmacology. 164, 71–77. 10.1016/j.jep.2015.01.048 25666427

[B21] Liu SN Sun SJ Liu Q Hou SC Shen ZF (2014). [Effect of Mudan Granule on Islets Beta Cell Function in Monosodium Glutamate Induced Obese Mice with Insulin Resistance: an Experimental Study]. Zhongguo Zhong Xi Yi Jie He Za Zhi. 34 (07), 853–8. 10.7661/CJIM.2014.07.0853 25137853

[B22] Liu YL Zhong YC He CH Zou BJ (2020). Clinical Observation on Treating Painful Diabetic Neuropathy with the Mudan Granules Plus Epalrestat with Mecobalamin. Clinical Journal of Chinese Medicine 12 (32), 71–4. 10.3969/j.issn.1674-7860.2020.32.026

[B23] Liu DS (1983). Pathophysiological Characteristics of the Elderly. Journal of Postgraduates of Medicine (03), 30–2.

[B24] Megallaa M. H. Ismail A. A. Zeitoun M. H. Khalifa M. S. (2019). Association of Diabetic Foot Ulcers with Chronic Vascular Diabetic Complications in Patients with Type 2 Diabetes. Diabetes & Metabolic Syndrome: Clinical Research & Reviews 13 (2), 1287–1292. 10.1016/j.dsx.2019.01.048 31336479

[B25] Ng C. Wang SP Cheong JL Wu YD Jia YL Leung SW (2012). Systematic Review and Meta-Analysis of Randomized Controlled Trials Comparing Chinese Patent Medicines Compound Danshen Dripping Pills and Di'ao Xinxuekang in Treating Angina Pectoris. J Chin Integr Med 10 (1), 25–34. 10.3736/jcim20120105 22237271

[B26] Peltier A. Goutman S. A. Callaghan B. C. (2014). Painful Diabetic Neuropathy. Bmj. 348, g1799. 10.1136/bmj.g1799 24803311

[B27] Saeedi P. Petersohn I. Salpea P. Malanda B. Karuranga S. Unwin N. (2019). Global and regional diabetes prevalence estimates for 2019 and projections for 2030 and 2045: Results from the International Diabetes Federation Diabetes Atlas, 9th edition. Diabetes Research and Clinical Practice 157, 107843. 10.1016/j.diabres.2019.107843 31518657

[B28] Tesfaye S. Selvarajah D. Gandhi R. Greig M. Shillo P. Fang F. (2016). Diabetic peripheral Neuropathy May not be as its Name Suggests. Pain 157 (Suppl 1), S72–s80. 10.1097/j.pain.0000000000000465 26785159

[B29] Li L Yu P (2016). Therapeutic Efficacy of Mudan Granule for 60 Patients with Diabetic Peripheral Neuropathy. International Society of Digital Medicine: The first academic exchange meeting of the digital Chinese medicine.(Guangdong, China: Zhuhai)

[B30] Wu Y (2021). Effect of Mudan Granule Combined with Routine Western Medicine on Nerve Conduction Velocity and Its Curative Effect on Diabetic Peripheral Neuropathy. Liaoning Journal of Traditional Chinese Medicine 48 (01), 112–5. 10.13192/j.issn.1000-1719.2021.01.032

[B31] Xun XY Liu SX (2020). Observation of Clinical Effect of Mecobalamin Combined with Mudan Granules in the Treatment of Type 2 Diabetic with Peripheral Neuropathy. Clinical research 28 (06), 108–9.

[B32] Yang LM Jia RM Gao Q Yin JD Li YZ (2020). Therapeutic Effect of Mudan Granule Combined with Mecobalamin on DPN ( syndrome of deficiency of Qi) and Collaterals and its Influence on Neuroelectrophysiology and Oxidative Stress Index. Journal of Clinical and Experimental Medicine. 19 (02), 181–4. 10.3969/j.issn.1671-4695.2020.02.019

[B33] Yau J. W. Y. Rogers S. L. Kawasaki R. Lamoureux E. L. Kowalski J. W. Bek T. (2012). Global Prevalence and Major Risk Factors of Diabetic Retinopathy. Diabetes Care 35 (3), 556–564. 10.2337/dc11-1909 22301125 PMC3322721

[B34] Zhang HQ Zhu HX (2020). Research on Clinical Effect of Xuesaitong Capsule Combined with α-lipoic Acid and its Effect on Serum TNF-α and IL-1β of Patients with Type 2 Diabetic Peripheral Neuropathy with Qi Deficiency and Blood Stasis Syndrome. Anhui Medical and Pharmaceutical Journal 24 (07), 1448–52. 10.3969/j.issn.1009-6469.2020.07.045

[B35] Zhao SH Jin D An XD Lian FM (2020). Experience in the Treatment of Diabetic Peripheral Neuropathy with Astragalus Root,Cassia Twig and Caulis Spatholobi——Three Prescription by Professor TONG Xiaolin. Jilin Journal of Traditional Chinese Medicine 40 (09), 1131–3. 10.13463/j.cnki.jlzyy.2020.09.004

[B36] Zheng Y. Ley S. H. Hu F. B. (2018). Global Aetiology and Epidemiology of Type 2 Diabetes Mellitus and its Complications. Nat Rev Endocrinol 14 (2), 88–98. 10.1038/nrendo.2017.151 29219149

[B37] Zheng YY (2002). Guidance Principle of Clinical Studies of New Drug of Traditional Chinese Medicine. Beijing, China: China Medical Science Press

[B38] Zuo JH Xiao JM Xie QW Chen J He YH Liu SN (2020). Network Meta-analysis on Clinical Effects of Six Blood-activating Stasis-removing Chinese Patent Medicines in Treatment of Diabetic Peripheral Neuropathy. Traditional Chinese Drug Research and Clinical Pharmacology. 31 (07), 867–73. 10.19378/j.issn.1003-9783.2020.07.018

