# Supplementary material for: Efficacy of Mudan Granule (Combined With Methylcobalamin) on Type 2 Diabetic Peripheral Neuropathy: Study Protocol for a Double-Blind, Randomized, Placebo-Controlled, Parallel-Arm, Multi-Center Trial
Source: Front Pharmacol. 2021 May 20;12:676503. doi: 10.3389/fphar.2021.676503 (PMC8173202; doi:10.3389/fphar.2021.676503)
Supplement: Supplementary file 2 [file Table2.DOCX]

**Michigan Diabetic Neuropathy Score (MDNS) Scale**

| **Clinical examinations** | **Scoring criteria** | **Score** | **Clinical examinations** | **Scoring criteria** | **Score** |
| --- | --- | --- | --- | --- | --- |
| **Sensory impairment** |  |  |  |  |  |
| Vibration at left big toe | 0: Normal  1: Decreased  2: Absent |  | Vibration at right big toe | 0: Normal  1: Decreased  2: Absent |  |
| 10-g filament at left big toe | 0: Normal  1: Decreased  2: Absent |  | 10-g filament at right big toe | 0: Normal  1: Decreased  2: Absent |  |
| Pin prick on dorsum of left great toe | 0: Painful  2: Not painful |  | Pin prick on dorsum of right great toe | 0: Painful  2: Not painful |  |
| **Muscle strength testing** |  |  |  |  |  |
| Left finger spread | 0: Normal  1: Mild to moderate  2: Severe  3: Absent |  | Right finger spread | 0: Normal  1: Mild to moderate  2: Severe  3: Absent |  |
| Left great toe extension | 0: Normal  1: Mild to moderate  2: Severe  3: Absent |  | Right great toe extension | 0: Normal  1: Mild to moderate  2: Severe  3: Absent |  |
| Left ankle dorsiflexion | 0: Normal  1: Mild to moderate  2: Severe  3: Absent |  | Right ankle dorsiflexion | 0: Normal  1: Mild to moderate  2: Severe  3: Absent |  |
| **Reflexes** |  |  |  |  |  |
| Left biceps brachii | 0: Present  1: Present with reinforcement  2: Absent |  | Right biceps brachii | 0: Present  1: Present with reinforcement  2: Absent |  |
| Left triceps brachii | 0: Present  1: Present with reinforcement  2: Absent |  | Right triceps brachii | 0: Present  1: Present with reinforcement  2: Absent |  |
| Left quadriceps femoris | 0: Present  1: Present with reinforcement  2: Absent |  | Right quadriceps femoris | 0: Present  1: Present with reinforcement  2: Absent |  |
| Left achilles | 0: Present  1: Present with reinforcement  2: Absent |  | Right achilles | 0: Present  1: Present with reinforcement  2: Absent |  |

**Toronto Clinical Neuropathy Scoring System Scale**

| **Symptom scores** | | | | **Reflex scores** | | | | | **Sensory test scores** | | | |
| --- | --- | --- | --- | --- | --- | --- | --- | --- | --- | --- | --- | --- |
| Foot | |  |  | Knee reflexes | | Left 0 | 1 | 2 | Pinprick | | 0 | 1 |
|  | Pain | 0 | 1 |  |  | Right 0 | 1 | 2 |  | Temperature | 0 | 1 |
|  | Numbness | 0 | 1 |  |  |  |  |  |  | Light touch | 0 | 1 |
|  | Tingling | 0 | 1 |  |  |  |  |  |  | Vibration | 0 | 1 |
|  | Weakness | 0 | 1 |  |  |  |  |  |  | Position | 0 | 1 |
| Ataxia | | 0 | 1 | Ankle reflexes | | Left 0 | 1 | 2 |  |  |  |  |
| Upper-limb symptoms | | 0 | 1 |  |  | Right 0 | 1 | 2 |  |  |  |  |

Sensory testing was performed on the first toe. Symptom scores: present = 1; absent = 0. Reflex scores: absent = 2; reduced = 1; normal = 0. Sensory test score: abnormal = 1; normal = 0. Total scores range from normal = 0 to maximum of 19.

**Nerve conduction velocity Checklist**

| **Nerves** | **Left** | | **Right** | |
| --- | --- | --- | --- | --- |
|  | **Nerve conduction velocity (m/s)** | **Assessment** | **Nerve conduction velocity (m/s)** | **Assessment** |
| Median sensory nerve |  | □normal □abnormal |  | □normal □abnormal |
| Ulnar sensory nerve |  | □normal □abnormal |  | □normal □abnormal |
| Sural sensory nerve |  | □normal □abnormal |  | □normal □abnormal |
| Superficial peroneal sensory nerve |  | □normal □abnormal |  | □normal □abnormal |
| Median motor nerve |  | □normal □abnormal |  | □normal □abnormal |
| Ulnar motor nerve |  | □normal □abnormal |  | □normal □abnormal |
| Peroneal motor nerve |  | □normal □abnormal |  | □normal □abnormal |
| Tibial motor nerve |  | □normal □abnormal |  | □normal □abnormal |

**Corneal Nerve Checklist**

| **Eye** | **Corneal nerve fiber density**  **no/mm^2^** | **Corneal nerve fiber length**  **mm/mm^2^** | **Corneal nerve branch density**  **no/mm^2^** |
| --- | --- | --- | --- |
| Left eye |  |  |  |
| Right eye |  |  |  |

**Clinical Symptoms Score of Traditional Chinese Medicine**

| **Primary symptoms** | **Scoring criteria** | **Score** |
| --- | --- | --- |
| Numbness | No numbness  Occasional numbness of the extremities  Continuous numbness of the extremities, limited to hands and feet  Persistent numbness below the knee or elbow | 0  2  4  6 |
| Pain | No Pain  Occasional stinging of extremities  Persistent pain in the extremities, but tolerable  Persistent pain in the extremities, affecting sleep | 0  2  4  6 |
| Paresthesia  (abnormal temperature sensation) | No paresthesia  Mild paresthesia  Moderate paresthesia  Intolerable paresthesia | 0  2  4  6 |
| Paresthesia  (formication) | No paresthesia  Mild paresthesia  Moderate paresthesia  Intolerable paresthesia | 0  2  4  6 |
| Paresthesia  (The paresthesia distribute like glove and sock) | No paresthesia  Mild paresthesia  Moderate paresthesia  Intolerable paresthesia | 0  2  4  6 |
| **Secondary symptoms** | **Scoring criteria** | **score** |
| Scaly dry skin | No symptoms  Limited roughness, dryness, loss of moisture  Rough and dry skin, keratinization, desquamation, flush at the base, melted into a piece  Extensively rough and dry, keratinization, and shaped like snakeskin | 0  1  2  3 |
| Dim complexion | No symptoms  Dark but not dirty and glossy  Dark, slightly dirty and slightly glossy  Dark and dirty, without glossy | 0  1  2  3 |
| Fatigue | No symptoms  Inclined to get tired, labor makes tired, but can stick to physical labor  Mental fatigue, activity makes tired, and barely support the daily work  Depressed, immobile, and unable to support daily activities | 0  1  2  3 |
| Spiritlessness  and talking laziness | No symptoms  Shortness of breath after the activity, do not like to talk much  Shortness of breath after a little activity, and lazy of speech  Shortness of breath when calm, do not want to speak, and torpid appearance | 0  1  2  3 |
| Spontaneous sweating | No symptoms  Slight sweating after the activity, and slightly wet clothes  Moisture of inactive skin, especially slightly moved  Sweating normally, a little activity makes sweating come out like water stains | 0  1  2  3 |
| Tongue and pulse | Tongue condition: light-dark/petechiae tongue, with thin and white coating  Pulse condition: thin and astringent pulse | Not scored |
